# Statistical methods for analyzing immunosignatures

**DOI:** 10.1186/1471-2105-12-349

**Published:** 2011-08-19

**Authors:** Justin R Brown, Phillip Stafford, Stephen A Johnston, Valentin Dinu

**Affiliations:** 1 Department of Biomedical Informatics, Arizona State University, Mayo Clinic - Samuel C. Johnson Research Bldg, 13212 East Shea Boulevard, Scottsdale, AZ 85259, USA; 2 Arizona State University - Center for Innovations in Medicine, Biodesign Institute and School of Life Sciences, 727 E. Tyler St. Tempe, AZ 85287, USA

## Abstract

**Background:**

Immunosignaturing is a new peptide microarray based technology for profiling of humoral immune responses. Despite new challenges, immunosignaturing gives us the opportunity to explore new and fundamentally different research questions. In addition to classifying samples based on disease status, the complex patterns and latent factors underlying immunosignatures, which we attempt to model, may have a diverse range of applications.

**Methods:**

We investigate the utility of a number of statistical methods to determine model performance and address challenges inherent in analyzing immunosignatures. Some of these methods include exploratory and confirmatory factor analyses, classical significance testing, structural equation and mixture modeling.

**Results:**

We demonstrate an ability to classify samples based on disease status and show that immunosignaturing is a very promising technology for screening and presymptomatic screening of disease. In addition, we are able to model complex patterns and latent factors underlying immunosignatures. These latent factors may serve as biomarkers for disease and may play a key role in a bioinformatic method for antibody discovery.

**Conclusion:**

Based on this research, we lay out an analytic framework illustrating how immunosignatures may be useful as a general method for screening and presymptomatic screening of disease as well as antibody discovery.

## Background

The human immune system is a rich source of information about the health and disease status of an individual [1-4]. Immunosignaturing is a new technology that may be useful to decode the vast amounts of health information contained in the immune system. An immunosignature is a pattern containing multiplexed signals from chronic or recently matured antibodies. These signals come from a sufficiently diverse set of peptide targets on a microarray. Thousands of peptides of random sequence (mimotopes) provide the density and diversity sufficient to discriminate different diseases. An initial question, and the aim of this paper, is how best to analyze and decode the information from immunosignaturing studies. Previous reports [1-3] used frequentist statistics (ANOVA or t-test) and cluster analysis (hierarchical clustering and Principal Components) to identify features that classify disease states. We examine other methods that may yield better performance in immunosignature analyses. Corrected T-Tests as well as logistic and multinomial logistic regression models have demonstrated an ability to differentiate between patients with different disease states even after stringent corrections for running multiple statistical tests (alpha inflation). Confirmatory factor analysis is an additional method which provides an abundance of information relating to the clustering of samples as well as providing an alternative method for categorizing and determining the disease state of a single sample. Descriptive statistics help to paint a better picture of the overall immune system activity. Finally, structural equation modeling and mixture models can help explain the underlying structure of an immunosignature.

For these analyses we examined a dataset containing breast cancer samples along with patients who had a second primary tumor (not a recurrence). The group with a second primary tumor was included in the analyses because if these patients could be diagnosed as having a high probability of developing a second tumor, they could be more closely monitored.

In an immunosignaturing study, sera samples are collected from participants and the physical information from the immune system is extracted using high density peptide microarrays. Each microarray contains a large number of peptides; in this case 10,375 peptides. The selection of these peptides was designed to give broad spectrum coverage of relevant antigens in the human immune system. The relevant nature of each peptide capitalized on early phage display research [1]. The decision was made to use a peptide microarray instead of phage library panning because of the increased speed and efficiency offered by a peptide microarray [1]. Ideally, if we can better understand the information captured by the peptide microarrays we may be able to develop quick, accurate, unobtrusive and inexpensive screening tests for many types of disease.

Classic peptide microarrays are created by spotting overlapping peptides corresponding to linear sequences of proteins known to be involved in an infectious disease. These arrays cannot identify non-linear epitopes. The epitopes are identified when B-cells produce antibodies (usually IgG) specific to 8-12 residue peptides that are components of the antigen protein. In contrast, immunosignaturing arrays utilize random-sequence peptides. Random sequence peptides have some specific and reproducible affinity to antibodies, and determining the level and pattern of binding is core to determining the difference between patients with different diseases.

Although much research has been done on statistical analyses using microarrays, immunosignaturing microarrays pose a number of novel challenges not encountered in traditional microarrays. In nucleic acid microarray technologies, binding is essentially only between two types of molecules of complementary sequence. For example, in a genotype array, genomic DNA binds to complementary nucleic acid probes that have either matches (e.g., perfect match, PM) or mismatches (MM) and the signals from the different probes are combined to make homozygous and heterozygous base calls for individual single nucleotide polymorphisms (SNPs). In a gene expression microarray, only a specific fragment of RNA will bind to the oligonucleotide on the array. With modern microarrays, as long as there is a sufficient abundance of RNA on the array, it will generally bind only to the specific complementary probe, with very limited non-specific binding.

With immunosignaturing microarrays, the intensity values are a continuous value from 0-65,000 and binding is not restricted to a single "complementary" molecule. Multiple antibodies in IgG could bind to the same 20mer peptide on the array. Also, although the immunosignaturing arrays are designed to measure IgG, there may still be competitive binding from other material in the sera and from other types of immunoglobulin. Competitive binding could result in an IgG antibody not binding at all or binding with a lower affinity. This could be potentially problematic if the auxiliary particle reducing binding affinity does not differ systematically across groups. Furthermore, a single antibody may also bind to multiple peptides on the array; a problem almost non-existent in genotype or gene expression arrays.

With the potential for so many different things to bind to a peptide on the array, it is not immediately clear how accurately traditional and more novel statistical methods would perform. One primary goal of the research reported here was to determine if the proposed statistical methods were capable of effectively analyzing the data and producing a correct pattern of results. For example, with a number of different things binding to a peptide and antibodies binding to multiple peptides it was initially uncertain if this would produce erratic signatures which would lead to incorrect results when certain methods were used.

Despite a number of new complexities created by immunosignaturing microarrays, these challenges give us the opportunity to test the performance of classically used methods such as factor analysis models in a different environment while also allowing us to ask new and fundamentally different research questions. In order to answer these new research questions, there is a need to use different statistical models not commonly used to analyze microarray data. This is because more traditional models used to analyze microarrays lack the versatility to adequately capture and explain the complexities of immunosignatures. Here, we explore the use of structural equation models in order to try to determine whether the immunosignature formed by the fluorescent values of the 10,375 peptides is mostly random or if there is a consistent underlying pattern or factor structure to an immunosignature that correlated with disease. This research question is made possible because of the novelty in immunosignaturing arrays that that allow a single antibody to bind to multiple peptides on the array. This research shows that there are complex and consistently reproducible structures underlying peptides which differentiate groups. Such patterns can be used as biosignatures for disease as well as provide deep insight into antibodies and immune response to disease. Although there are new analytic challenges in immunosignaturing, it is these exact challenges that provide the promise of new discoveries while laying the groundwork for applications in future research and technologies.

In this paper we present a range of statistical methods, their use and demonstrate what type of information they can provide researchers in immunosignaturing studies. We show the ability to classify samples into their respective disease categories and find peptides which significantly predict disease status. This provides a promising method for screening and potentially presymptomatic screening of disease. We also identify a number of latent factors using SEM. We hypothesize that the latent factors being modeled may represent specific antibodies that differ among disease classes.

## Methods

Patient samples are analyzed by applying the sera or plasma to the array at a 1:500 dilution, detected with an anti-human fluorescent antibody, and the signals are read using an Agilent C laser scanner. Images are processed using GenePix Pro 8 providing a text file of values for each peptide. Binding affinity is a continuous value from 0-65,500 (16 bit image). Genepix software was used to convert the 16-bit TIFF images to values, median non-background subtracted values were used and log_10 _transformation was done on the median normalized intensity values. Three distinct datasets were used in these analyses. One was a set of samples from a random group of individuals without breast cancer, a second set of samples is from a group with breast cancer and finally the third set of samples is from a group of patients who were diagnosed with a second primary tumor. The normal samples were a convenient sample of individuals without any known breast cancer history. The breast cancer samples were a sample of current breast cancer patients with different levels of disease progression and diverse demographic backgrounds. There were 52 samples from normal individuals without cancer, 98 samples from cancer patients with a single primary tumor and there were 21 samples with second primary tumors. Human subjects protection was observed, collaborators ensured all samples were collected under the same protocol. All of the sample came from females between the age of 45 and 54. The specific ages for each sample was kept from us because of HIPPA and patient privacy concerns. All pre-processing was median-normalization per microarray slide, to adjust for global intensity bias. Data was also log_10 _transformed. The spot intensity was the median signal (obtained by GenePix Pro) with no local background subtraction. Background subtraction was not used because the arrays showed consistent background across the 1172 empty spots which were spread across the physical surface of the array. Technical replicates also showed greater reproducibility without background subtraction than with, indicating that the method for subtracting background was not useful. Additionally, the local and global background estimates were, on average, 150-300 RFU, which for any microarray is extremely low considering the 3+ logs of dynamic range.

It is common in similar lines of research, such as genotype experimentation to use a pattern matched experimental design. Matching participants in an experiment has the effect of increasing homogeneity among groups. As a result, the reduced within class variation which often accompanies matching designs has the effect of reducing the standard error and denominator of common statistical tests. This in turn leads to higher statistical power. Additionally, more homogeneous groups often enable easier classification in exploratory models. In the data analyzed here, the normal non-cancer samples were not matched to either the cancer groups, however research has shown that the signature of immune response is far less susceptible to the type of personal factors that genetic studies are - even HLA has only a minor effect on the consistency of a disease state immunosignature pattern [1,2].

Given that immunosignaturing is a new technology, early investigations, contrary to initial belief actually capitalize on the lack of rigid experimental designs. This is because additional sources of variance in the data allow us to better understand the robustness of the technology and related statistical analyses. If a method can perform well in a somewhat noisy environment with loose experimental designs, it is highly likely to perform even better when well curated studies (such as matched designs) are performed. In many respects, testing immunosignaturing data with loosely structured and curated data provides a much more stringent test of the technology and methods. Being able to obtain statistically significant results with the correct patterns of results from such unstructured data illustrates the versatility of immunosignaturing technology and the statistical methods tested here.

Understanding the robustness of the technology provide guidance for future experiments using this technology while giving insight into the potential clinical use of immunosignaturing. Biologically, it is possible that healthy normal individuals with no active infection are responding immunologically to their environment, and persons with an infection have a focused immune response. It is likely that high variation in immune response to an environment would be present across individuals. Therefore, in order to be clinically useful, it is imperative that the technology and methods are robust enough to function accurately outside of precisely controlled laboratory settings; as would be encountered during clinical deployment of the technology.

## Results

### Descriptive Statistics

Table 1 provides basic descriptive fluorescence intensity statistics of each of the three disease groups. Descriptive statistics of an immunosignature provide a significant amount of insight into the underlying immune response during disease states. Of particular biological interest in this sample is the difference in the range of values from the three groups. The normal and single tumor cancer samples have ostensibly the same floor value while the second primary tumor cancer samples have a much lower floor value. This may suggest a suppression of the immune system in second primary tumor cancer samples. The single tumor cancer and second primary tumor cancer samples have progressively higher maximum values which may suggest an increased immune response associated with cancer and a reoccurrence of cancer.

**Table 1:** Fluorescence intensity and descriptive statistics for the three disease groups

Group	Mean	Minimum	Maximum	Std. Deviation	Variance	Range
Normal	329	207	9672	93	10336	9465

Single Tumor	336	204	16702	115	16258	16498

Second Tumor	676	36	49880	549	339301	49844

Although there are large differences in the ranges, in order to have any predictive validity, the differences in ranges need to be consistent across samples within each group. For example, a high fluorescence value over 45000 in the second tumor samples needs to occur on a given peptide with regularity to produce a statistically significant result.

### Classical Statistical Significance Tests

There are a number of statistical tests which could potentially be used to test whether the differences between groups across peptides are significant beyond what would be expected by chance alone. Some of these methods include the T-Test, corrected T-Tests, Logistic Regression and Multinomial Logistic regression. The standard T-Test divides the mean difference between two groups by a standard error to produce a T-Statistic used for null hypothesis significance testing. One problem with the standard T-Test is that normal theory underlying the test makes the assumptions that the variances in both groups are equal. The problem of unequal variances in a T-Test is commonly known in the statistics literature as the Behrens-Fisher problem and has been researched for the better part of the last century in various contexts. If the assumption of equal variances is violated, the T-Statistic can be either inflated or deflated depending on the samples sizes in each group. As a result, the analyses were conducted using a Satterthwaite corrected T-Test. The Satterthwaite test is one of numerous corrections for unequal variances that have been proposed over the years. The Satterthwaite test works by adjusting the degrees of freedom in the test. The resulting correction produces an asymptotically correct T-statistic when groups have unequal variances. The Satterthwaite correction works by modifying the degrees of freedom via equation 1:

(1) $$df=\frac{\left(w1+w2\right)*2}{\begin{array}{ccc} \frac{w1*2}{n2-1} & + & \frac{w2*2}{n2-1} \end{array}}$$

A Satterthwaite corrected T-Test and a number of similar test corrections which could have also been used such as a Brown-Forsythe correction in an ANOVA model tended to produce statistically significant results after a Bonferroni correction for multiple testing (alpha inflation). A Bonferroni correction was used to protect against alpha inflation because with a standard alpha level of .05, purely by chance alone, 1 out of 20 tests will be significant. The Bonferroni correction divides the alpha value by the number of tests run; in this case 10,375, or one for each peptide on the microarray. This resulted in a corrected p-value threshold of 4.819*10^-6^. Nonetheless, despite this much lower p-value, highly significant results are still obtained for Satterthwaite corrected T-Tests comparing normal versus single tumor cancer samples, normal versus second diagnosis samples and single tumor cancer versus second primary tumor cancer samples. Table 2 shows the top 10 significant peptides for a Satterthwaite corrected T-Test comparing normal samples to cancer samples. Logistic and Multinomial logistic regression may also be of interest and an alternative method for comparing groups to the tests used here. One place in which logit models may be useful is if a researcher in future studies has a known set of covariates they wish to control for. For example, in the study of diabetes, it may be of interest to control for body mass index or HB1AC test results.

**Table 2:** Top 10 significant peptides for a Satterthwaite corrected T-Test comparing normal samples to cancer samples

Variable ID	Peptide Sequence	T-Value	Degrees Of Freedom	P-Value
V2833	HFRKWHKRRWKHHKKWKGSC	-6.51	132.4	1.4372E-09

V3113	HRFKWHWKHRFHHFHRFGSC	-6.29	144.41	3.5843E-09

V6772	QKFKHQQGSFKLPWLSMGSC	-6.29	144.84	3.5843E-09

V9732	WRRSTPVGPWTWFGKFLGSC	-6.12	146.1	8.1933E-09

V7196	RFGRPQHQHDFRRHAIYGSC	-6.06	146.8	1.1046E-08

V6978	QSHMTLAPGIRRYKKFNGSC	-6.06	146.32	1.1046E-08

V7387	RMGFGLYERLWGKTNHYGSC	-6.01	134.26	1.6532E-08

V9561	WKWKRHWKWPHRRKHFFGSC	-5.95	144.49	1.9475E-08

V6987	QSIGLGYSAFMPKWPFRGSC	-5.93	140.13	2.2543E-08

V3249	HWKRHHRPKHKHHRHKHGSC	-5.9	145.4	2.4586E-08

### Exploratory Factor Analysis

Factor analytic models have previously been used in analyzing immunosignatures and are quite common in analyzing high dimensionality microarray data [2,5,6]. Each of the models explored during this line of research were investigated in order to determine its feasibility for answering a specific research question. Exploratory factor analysis (EFA) was examined as a method to be able to differentiate samples based on disease states with no prior clinical knowledge of the samples. Estimation of EFA models was performed using ordinary least squares (OLS). EFA with Promax rotation proved significantly better than chance at classifying samples. EFA is a set of procedures that accounts for the relationship among a set of variables in terms of a smaller set of underlying latent constructs or factors. (For example, a factor is a disease state.) We specifically use principal axis factoring with iterated communalities. Although PCA and EFA are quite similar, an important difference between the two methods is that PCA makes the assumption that all of the variance in an item is a reflection of common variance shared among all items whereas EFA posits that each item shares some common variance with all other items but also has its own unique variance. Mathematically the difference between PCA and EFA is the addition of single matrix; D^2^.

(2) $$\text{Rzz}=\text{A}\;*\;\;\text{Rf}\;*\;\text{A}\text{’}+{\text{D}}^{2}$$

In equation 2 R_zz _is the correlation matrix among the observed variables. A is a matrix of factor loadings, R_f _is the correlation matrix among the factor loadings, the A' denotes the transpose of the A matrix of factor loadings and thus AR_f_A' is the matrix representation of the common factor structure. D^2 ^is a diagonal matrix that captures the unique variance weights and distinguishes EFA from PCA.

Varimax and Promax rotation methods were explored in depth. This is in part because Varimax is often a starting point for a Promax rotation. A sample is said to "load on" a given factor when the model suggests a strong fit on the given factor. Rotation in EFA is a method for making factor loadings more interpretable. Rotation methods change the relationship between items and the factors (which are geometrically represented as axes). Rotation does not change the relationship among the individual items. Since rotation methods only make changes to the axes and not to the communalities (variance accounted for), rotation does not mathematically change the initially obtained results. Rotation makes the factor loadings more interpretable.

Varimax uses a complexity function to maximize the variance of the squared loadings on each factor. This results in loadings with a more even spread across the factors; as opposed to having an overabundance of loadings on a first factor. Varimax is an orthogonal rotation that maintains the orthogonal (90 degrees) intersection of the axes. This has the result of keeping the correlation between the factors at zero because the cosine of 90 degrees is 0.

Promax is an oblique rotation that allows the angle between the axes to vary. In statistics, variance has to be accounted for in some part of the model. Allowing the axes to vary and thus a correlation between the factors is another path to account for variance. Allowing variance to be expressed in terms of correlations between factors has the result of not forcing variance between factors to be represented as between item variance. This can result in cleaner factor loadings. Additionally, the assumption that there is no correlation between factors, or in this analysis, disease states, is unlikely because there will always be some additional common variance and similarities in immune samples due to basic immune responses and structures present across all samples.

Unlike Varimax, Promax does not use a complexity function. Rather, Promax rotation is a procrustean rotation to a target matrix. In Promax, a pattern matrix of loadings (often derived from Varimax rotation) is taken to some power (i.e., squared, cubed etc.) to form a target matrix. The original loading components are then rotated to get as close as possible to the newly formed target matrix. A number of EFA models with Promax rotation were run to investigate the utility of this method for differentiating between groups with no prior knowledge of group membership. Table 3 provides summary results. The number of factors was known to be 2 for each comparison. Scree plots were used to validate the hypothesis. None of the plots suggested the presence of a strong third factor. A scree plot plots the eigenvalues for each component. The largest components before a leveling off is used to determine the appropriate number of factors. Factor loadings greater than .3 were said to load on a given factor. If loadings for both factors were less than .3 the sample was said to not counted as a correct classification on either match. Catell (1966) provides a more detailed description of how to use eigenvalues and scree plots for determining the number of factors [7].

**Table 3:** Exploratory Factor Analysis Results

EFA Model	Correct Classification
Single Primary Tumor and Second Primary Tumor Samples	93.45%

Non-Cancerous and Second Time Cancer Samples	84.4%

Non-Cancerous and First Time Cancer Samples	68%

An EFA between cancer samples and the samples from patients who had a second primary tumor produced a correct classification for 93.45% of the cases. Of the cases that were miscategorized, all of them except one were cancer cases that loaded more highly with the second primary tumor group. There are a few possible explanations for this. This could simply be model error resulting from the lack of homogeneity among the first time cancer group. However, it is possible that the miscategorized cases may represent individuals who will at some point in the future develop a second primary tumor or are unbeknownst to the researchers already in the process of developing one. All this says is that less than 10% of cancer samples are more closely related to the samples of individuals who had acquired a second primary tumor than the samples with a single primary tumor.

A second EFA was run between the normal or noncancerous samples and the samples with a second primary tumor. The overall classification accuracy was 84.8%. Within this model, 74.1% of the normal samples loaded correctly on the same factor whereas 100% of the second primary tumor samples loaded on the correct and same factor.

A third EFA was run exploring the relationship between normal or non-cancerous samples and single tumor cancer samples. Using the same model specifications as in the first model, this EFA produced a 68% classification accuracy. Although this is quite low by traditional model building standards, there are a number of factors relating to the data which may make this a useful starting point. First, the normal patients were taken from a wide range of convenient lab samples. Some of the normal samples may have come from individuals outside of the age and traditional demographic background to even be remotely at risk for breast cancer. Secondly, the stage and progression of cancer patients was unknown. As a result, an additional possibility for the classification accuracy may be that the cross loadings represent a mixture of early stage cancer patients and those at high risk for or who are developing cancer.

Unfortunately, detailed information about the disease state of the samples is unavailable and does make conjectures purely hypothetical. However, in all models, the results are significantly better than chance and illustrate in many ways the performance of the technology and approach under adverse conditions. The three models taken in concert illustrate that the lack of a concrete and well curated control group is likely responsible for the decremented classification accuracy in some models. This can be most clearly seen when considering that the single tumor cancer and second primary tumor cancer samples consistently exhibit stable factor loadings with relatively low cross loadings because the single tumor cancer samples serve as a much cleaner control group for the second primary tumor cancer samples than the normal do for either of the cancer groups. This early research suggests that future studies using more precisely selected control groups and experimental design would have even better ability to classify cancer patents.

Beyond classification accuracy, the similarity between different factor based models and rotations is extremely informative from a biological perspective. All combinations of PCA and EFA with Varimax or Promax gave highly similar results with respect to overall classification of groups across a number of different analyses. Although specific factor loadings certainly had different values, the overall picture and classification accuracy was relatively constant. Brief investigations into other rotations such as Oblimin were also explored in the context of EFA models and produced similar results to Varimax and Promax.

First, with respect to PCA versus EFA, the lack of difference suggests that the vast majority of the variance accounting for classification is at the factor level (ie. ostensibly disease state) and not the individual level. This is because as the D^2 ^matrix which differentiates the two methods captures the unique variance in an EFA model and as the D^2 ^matrix approaches zero, an EFA model approaches a PCA model. Therefore, since the D^2 ^matrix is the only difference in the equation and an analytic solutions exists due to Ordinary Least Squares estimation, we can conclude that the lack of difference was because there was relatively little unique variance present.

### Confirmatory Factor Analysis

Since EFA models showed the ability to differentiate samples, a logical clinical application of immunosignaturing would be to screen a single sample from an individual to determine his or her disease status. Confirmatory factor analysis (CFA) was chosen as an ideal method for investigating this question due in part to the similarity with EFA and because of the versatility to examine one specific sample in detail. EFA is an exploratory method that should be used when the number of groups or structure of the data is not well understood. Conversely, CFA is a confirmatory method that can be used when the structure of the data is well understood. As the name implies, exploratory factor analysis, EFA models should not be used as confirmatory model or to confirm a hypothesis.

Both CFA and EFA attempt to explain the underlying structure in a dataset. However, CFA and EFA approach the problem from two distinct directions. EFA makes almost no prior assumptions about the structure of the data and attempts to sort through the data to help a researcher determine what the underlying structure of the data is. In this research, the general group membership was known and thus the appropriate number of factors was specified apriori. In a CFA model, the researcher explicitly identifies not only the number of factors but which cases load on each factor as well as factor variances, covariances between the factors and disturbances for each item. CFA models are not data mining approaches and require well formulated notions about the underlying structure of the data.

Mathematically, the simplest formulation of a CFA model in matrix notation is:

(3) $$\text{X}=\wedge *\;\xi *\;\Delta_{\text{L}}$$

In Equation 3, × is a vector of observed variables, Λ is a matrix of factor loadings, ξ is a matrix of scores for each variable on a factor or latent construct and Δ is a vector containing measurement error.

In the CFA models analyzed here, one sample from each factor (disease state) was chosen at random as a scaling constraint in order to ensure identification in these models. Maximum likelihood estimation with robust standard errors was used to estimate these CFA models. The known disease status was the basis for defining the factor loading for each sample. A sample was allowed to load only on a single factor and fixed to zero on the other. Variances and covariances between all factors were estimated. Summary results are provided in Table 4.

**Table 4:** Confirmatory Factor Analysis Results

CFA Model	Correct Classification
Single Primary Tumor and Second Primary Tumor Samples	89.9%

Non-Cancerous and Second Time Cancer Samples	93.1%

Non-Cancerous and First Time Cancer Samples	83.4%

For a CFA comparing single tumor cancer samples and second primary tumor samples, 89.9% of samples loaded on the specified factor. For a normal versus second primary tumor CFA, 93.1% of the samples loaded on the specified factor and a normal versus single tumor CFA produced sample loadings on the specified factor 83.4% of the time. The difference in classification accuracy between the CFA and EFA models is due to a number of factors; some of which include model variance and covariance specifications as well as different estimator types.

One primary advantage CFA models have over EFA models are fit indices which give some quantitative measure of how accurately the specified model is. Although there are a plethora of fit indices that have been proposed within the structural modeling framework that CFA models reside, the chi-square difference test, root mean square error (RMSEA) and standardized root mean error (SRMR) are among the most common and widely cited.

The chi-square test ostensibly tests how well the specified model reproduces the covariance matrix from the original data. The problem with this test is that it is so sensitive that it is nearly impossible to obtain statistically non-significant results. It is important to note that the null hypothesis of this test is that there is no difference between the specified model's covariance matrix and the covariance model in the actual data, a non-significant p-value is the desired outcome. Because it is of interest to find no difference between the specified model and the data, a non-significant p-value is the goal. The chi-square test for all of the CFA models was significant with p < .001 suggesting that there is a statistically significant difference between the specified model covariance matrix and the covariance matrix of the original data. However, the chi-square test is extremely sensitive and often detects trivial differences [8,9]. Noting the sensitivity of the test is not meant to suggest that in fact the specified CFA models are perfect fits or deny lack of fit. Rather, the test is noted because it is among the most common fit indices and the issues with the test are noted as a means of providing appropriate context for the results.

The Root Mean Square Error of Approximation (RMSEA) and the Standardized Root Mean Square Residual (SRMR) are two common fit indices used in the Structural Equation Modeling (SEM) framework description; of which CFA is a part of. The basis of the RMSEA is a non-centrality parameter. The simplest reduced form of the RMSEA equation is:

(4) $$\text{RMSEA}=\sqrt{\frac{\left(\frac{x^{2}}{df}\right)-1}{n-1}}$$

In equation 4, X^2 ^is the model generated chi-square value, df is the degrees of freedom and n is the sample size. Smaller RMSEA values suggest better fit. The SRMR measures the standardized difference between the observed covariance matrix and the model implied covariance matrix.

For the CFA model for single tumor samples versus second primary tumor samples, the RMESA was .083 and the SRMR was .071. For the CFA model comparing normal versus second primary tumor samples the RMSEA was .097 and the SRMR was .076 while the normal versus single tumor samples produced a RMSEA of .07 and a SRMR of .074. These are marginally significant results because traditional benchmarks cite .05 as a cutoff for statistical significance [8]. RMSEA and SRMR values in the .05-.08 range are usually regarded as marginally significant. Although the results do not meet the rigid .05 level, they are actually quite impressive when considering the experimental design and the fact that a portion of the lack of fit may actually be representing natural biological patterns such as the development of a first or second tumor.

Perhaps the real utility of a CFA model for immunosignaturing could come in the form of diagnostic testing. Given the accuracy of the CFA model with this data, once a well curated set of samples for a certain disease or collection of diseases has been established, a CFA model could be specified where a new unknown sample could be allowed to load on both (or multiple) factors. By comparing the relative loadings on the factors, it would be possible to determine to which group the sample most likely belongs. For example, there are numerous subtypes of breast cancer and different stages of disease progression. If a collection of samples was available as a concrete reference set, a CFA model could be easily and accurately employed as a new method for aiding in the diagnosis as well as perhaps early detection of breast cancer.

### Structural Equation Models

From EFA, CFA and descriptive statistics we know that the immunosignatures as a whole are in fact different across groups while corrected T-Tests show that there are statistically significant systematic variations. The logical question arising from these findings is how precisely do the immunosignatures differ from one another? Is there a clear, consistent and reproducible pattern underlying the differences in immunosignatures across disease states? Because a single antibody can bind to multiple peptides and different antibodies can bind to the same peptide, a coherent pattern of peptide fluorescence across an immunosignature is much more informative than the fluorescence of individual peptides on their own. Furthermore, being able to identify common relationships and covariances between groups of peptides is of even greater utility. This can be accomplished by modeling latent factors.

On a genotype microarray, the probe is directly measuring an individual's genotype at a specific location. In contrast, the peptide probes on an immunosignature array are indirectly measuring immune response and antibodies present in the sera. When measures are not directly observed they are often referred to in statistical and structural equation modeling literature as latent factors. If there are clear, consistent and reproducible patterns caused by specific antibodies in a sera sample binding to peptides on an immunosignaturing array, it should be possible to model individual antibodies as latent factors. For example, when reading the tick marks on a mercury thermometer, one is not reading a direct measure of temperature but rather displacement of mercury. The latent factor measured by displacement of mercury is temperature because from a purely physics standpoint, temperature is the kinetic energy of an object; usually measured at the molecular level. Another example of a latent factor is depression. Psychologists cannot directly measure depression but they can ask a series of questions that cumulatively allow them to model the latent construct of depression. Each question in a depression inventory gets at one small piece of the latent factor depression in much the same way that peptides on an immunosignaturing array provide an indirect measure of immune response; as measured primarily by IgG antibodies.

Structural equation modeling (SEM) is specifically designed for modeling latent variables. SEM models have two parts: a path model comprised of regressing a set of variables on another and a measurement model in which CFA is used to form latent variables. When a set of measured variables is set to load on a given factor, the result is a latent factor. In SEM, the resulting latent variables can be treated as either endogenous or exogenous variables; depending on the research question of interest. A full SEM model is a collection of equations defining each variable and their relation to one another. Since complex models can quickly generate a large number of equations, SEM models are often represented graphically for quicker interpretation. Since confirmatory factor analysis is a major component in a full latent variable structural equation model, attempting to classify samples with factor analytic methods lent evidence to the feasibility of SEM models. These early models also provided a plethora of background information which aided in the testing of full SEM models.

### Initial SEM Testing

Despite evidence from previous factor analytic models that SEM models should be feasible, since these are highly complex models, an incremental approach was taken to building and testing large scale SEM models. To start with, a measurement model and full structural equation model was run using the top three peptides from the normal versus single tumor cancer samples (Table 2) to predict disease. The measurement model (ostensibly a confirmatory factor analysis) in a SEM model tests the loadings of individual peptides onto latent variables. In this model one peptide was set as a scaling constraint and the other two were freely estimated. Three peptides were chosen because that is the minimum needed for model identification and provides for the simplest model. Because of the iterative nature of the maximum likelihood algorithms used in SEM models, starting with a simple model reduces computational time and aids in convergence. Furthermore, starting with the simplest model and building up is good practice in modeling.

Since a measurement model with 3 factors is just identified or has no extra degrees of freedom, fit indices cannot be calculated. However, all the variables load strongly on the latent factor with loadings greater than .7. This finding suggests that the top 3 peptides are indicative of a single underlying latent factor.

In order to help rule out the possibility that the consistent loadings in the first model were not type 1 error or false positive, the same model specification was run in an attempt to see if the top 3 peptides differentiating single tumor cancer samples from second primary tumor cancer samples. In this model the top 3 peptides also loaded on a single latent variable. Like the first model, the second model illustrated the same pattern of results with the top 3 peptides all significantly loading on a single latent factor.

The same pattern of results can be replicated with two disease contrasts. Replicating the finding with normal versus a single primary tumor cancer and second primary tumor cancer versus single primary tumor dramatically reduces threats to validity against causal conclusions proposed by SEM models of immunosignaturing data.

When investigating models that differentiate two distinct groups from a baseline group (in this case single tumor cancer samples) there are three potential outcomes. First, a complete lack of model fit and no consistent underlying factor structure. In this case, none of the peptides would load consistently and correctly on either of two specified factors suggesting that peptide florescence is random. The second possibility is that all of the peptides would load on one factor. This result could result from any number of potential biases in the technology itself, printing or processing of the microarrays. Another reason all of the peptides might load on one common factor is that they are all part of a single latent factor. However, because the significance of each peptide varies quite precipitously across group contrasts, it seems unlikely that a single underlying latent factor would produce different significance values across disease contrasts. The third possibility is that the peptides significantly load on two separate factors and that the peptides for each contrast exhibit no cross loadings.

**Figure 1:**
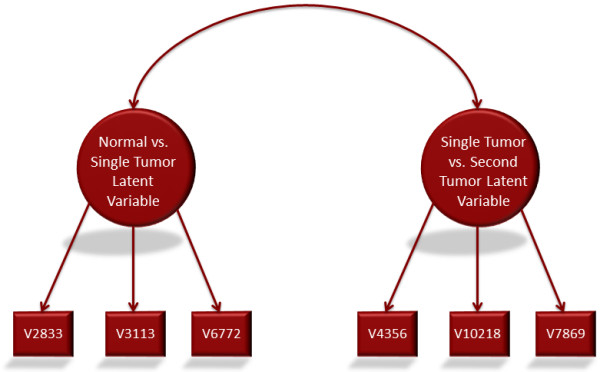
**Latent & measured variables in immunosignaturing: See Table 2 for peptide sequences**.

A series of analyses was run using significant peptides from normal versus single tumor cancer corrected T-Tests as well as second primary tumor samples versus single tumor samples combined into a single model. The first model was a measurement model which added the first two CFA's into one model. The top 3 peptides for normal versus single tumor samples and single tumor samples versus second tumor samples each were set to load on a separate latent factor. A covariance between the two latent variables was also estimated. The path diagram in Figure 1 illustrates this model. In Figure 1's path diagram, the square boxes represent measured variables, which, in this case are peptide fluorescent values. The large circles are the unmeasured latent variables. The arrows between the latent factors and measured variables show which measured peptides load on which latent variable. The curved arrow represents an estimated covariance between the two latent variables.

In path diagrams, the arrows represent the causal flow of information. The arrows are pointing from the latent variables to the measured variables because the argument in SEM models is that there is some unmeasured and underlying latent construct that is responsible for the observed results of the measured variables. The immune response and antibodies present in the sera samples is the ultimate causal factor of peptide fluorescence.

The model tested in Figure 1 was estimated using maximum likelihood estimation with robust standard errors (MLR). The model exhibits excellent model fit with an RMSEA of .063 and an SRMR of .031. In addition, the Chi-Square test was not significant, Chi-Sq = 14.054, df = 8, p = .0804. A non-significant Chi-Square test is the desired result. Again, this is because the null hypothesis of this Chi-Square test is that there is no difference between the observed covariance matrix (input data) and the covariance matrix implied by the model in Figure 1. These results strongly suggest excellent model fit and that the latent factors are unique constructs. Biologically, this suggests that a different latent factor is underlying each latent variable.

To further confirm the interpretation that the latent factors are different, one peptide from each factor was switched. V3113 and V10218 were set to load on the opposite factor from the first model. In this new model, there was a complete lack of fit. In addition to poor loadings, the fit indices dramatically decreased. The RMSEA was .354, the SRMR was .192 and the Chi-Square was 198.704, df = 8, p < .0001. Thus further suggests two different underlying constructs rather than statistical anomalies.

An additional set of analyses were run using the top 5 peptides instead of just the top 3. The first models run in this sequence were Varimax and Promax exploratory factor analyses. Both models gave 100% classification with extremely strong loadings on each factor. Table 5 is the rotated factor pattern or a two group EFA taking the top 5 peptides from each disease contrast. This clearly illustrates the top five peptides strongly load on factor one while the last five strongly load on the second factor. The loadings of peptides are consistent with the groups from which each peptide was selected. For example, v4356, v10218, v7869, v8672 and v8170 were the top 5 most significant peptides differentiating first time cancer samples from second time cancer samples. In combination with earlier results, this very clear and consistent loading pattern strongly suggests that the top peptides for each class form unique latent variables and they are almost irrefutably measuring different constructs. Biologically, this suggests that the latent factor which is more active in single tumor cancer samples compared to normal samples is not the same latent factor that appears to be present in second tumor samples.

**Table 5:** Rotated factor pattern for two group EFA of significant peptides

Peptide ID	Peptide Sequence	Factor1	Factor2
V4356	KYQFAGQRSGKQYRWRIGSC	0.88773	0.05624

V10218	YQPPPRKAVIQMDWLSYGSC	0.92126	0.06844

V7869	SKFRDVLTFNEPSRFVSGSC	0.51657	0.04716

V8672	TVHESMIYRMRFMTFKHGSC	0.93261	0.04783

V8170	SWRRMRMHKNFMISNLDGSC	0.87997	0.06368

V2833	HFRKWHKRRWKHHKKWKGSC	0.11128	0.7436

V3113	HRFKWHWKHRFHHFHRFGSC	0.06271	0.82673

V6772	QKFKHQQGSFKLPWLSMGSC	0.12145	0.73203

V9732	WRRSTPVGPWTWFGKFLGSC	0.05844	0.88795

V7196	RFGRPQHQHDFRRHAIYGSC	0.035	0.88098

The same result was also found by running a two group exploratory factor mixture model with Geomin rotation. Geomin rotation is another oblique rotation method similar to Promax. A more complete discussion of the mathematical differences of rotation methods can be found in Browne (2001) [10]. In this data, the observed peptides as a whole form a single distribution. In mixture modeling, the underlying notion is that the distribution formed by all of the observed data is the product of two or more underlying distributions; each of which represents a distinct class. Ostensibly, an exploratory factor mixture model is trying to answer the same question as PCA and EFA, PAF/Factor Analysis but via a different mathematical framework. Despite the complexity of mixture modeling, the basis of an exploratory factor mixture model is for a categorical latent class variable C, for a specific class k. The model estimated is:

(5) $${\text{Y}}_{\text{p}}=\;{\text{V}}_{\text{kp}}+\lambda_{\text{kp}}*\eta *\epsilon_{\text{p}}$$

In equation 5, for a variable Y_p_, V_kp _is an intercept parameter, λ_kp _is a vector of loadings, η is a vector of latent factors and ε_p _is a residual term. In addition, there is a correlation matrix Ψ_k _for the latent factors η of class k along with a distribution for the latent class variable C: P_k _= P(C = K). In this equation, for a dependent variable P, the probability of C is equal to k. Also, other constraints are added to this basic framework for purposes of identification but are related to model specific decisions such as orthogonal or oblique rotation.

EFA mixture models were estimated using Maximum Likelihood with Robust Standard Errors (MLR) estimation and 20 random start values. Random starting values were used in part due to the complexity inherent in mixture models and to check for local solutions. By running the analysis with multiple random start values log likelihood (LL) values can be compared. To the extent that different LL values are obtained, the random start values can be directly input into the model and the results can be compared to the best fitting LL model. This is useful because if different start values produce dramatically different results, this might suggest that the algorithm converged at a local maxima instead of global maxima or that the results are unstable.

Fit statistics such as the Bayesian Information Criterion (BIC) provide a more quantitative analysis of model fit for a series of nested models. EFA mixture models were estimated for one, two and three class models. This approach allows us to confirm that a two class model is in fact the best fit for the data.

The series of EFA mixture models suggested the same pattern of results as traditional EFA models; that there are two distinct and separate underlying classes formed by the top 5 peptides for each disease contrast. In addition, mixture models also produce a statistic for the average latent class probability:

(6) $$\text{P}\left({\text{Y}}_{\text{p}}=\text{j}|\text{C}=\text{K}\right)=\varphi -\text{1}\left(\text{T}*\text{kpj}\right)-\varphi -\text{1}\left(\text{T}*\text{kpj}-\text{1}\right)$$

In equation 6 T*_kpj _is a threshold parameter on a standardized correlation metric and φ is a matrix of residuals for the latent factors [11]. For both two and three class models, the average latent class probability for the most likely latent class membership was greater than 99% for both class 1 and class 2. In other words, for the subgroup of samples classified as being part of class 1 by the model, more than 99% of the time, class 1 was also their most likely class membership. This further reaffirms the excellent model classification. The three class model produced nearly identical average latent class probability values because the model did not classify any of the peptides as belonging to the third class.

The BIC was used to assess the best fitting model. The BIC is estimated as follows:

(7) $$\text{BIC}=-\text{2}*\text{LL}+\text{p}*\text{log}\left(\text{n}\right)$$

In equation 7 LL is the log likelihood value of the model, p is the number of parameters and n is the number of observations. The lower the value of the BIC the better the model fit. Often times, BIC values or plots are used ostensibly in the same fashion that scree plots and eigenvalues are used in PCA or traditional factor models where a researcher looks for the point at which the decrease in values levels off. However, in this analysis, the two class model had the lowest BIC and somewhat unexpectedly, the three class model actually saw a slight increase in the BIC This result further reaffirms the excellent fit of a two class model.

**Figure 2:**
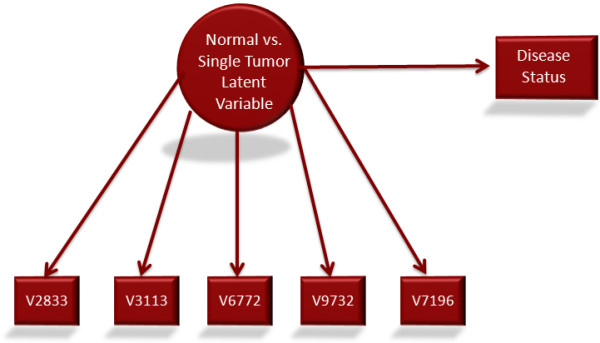
**Path diagram for normal versus cancer peptides SEM model**.

As is common in model building, a series of full structural equation models (SEM) were run in increasing levels of complexity. To start with, the two latent variables were regressed on their respective disease states in individual models. A path diagram for the normal versus single tumor samples is presented in Figure 2.

These models were estimated using MLR. The latent variable regression was performed using logistic regression and was significant, p < .001. Additionally, the odds ratio was 1.841. This suggests that having the attributed measured by the latent variable makes an individual 1.841 times more likely to develop breast cancer. The same model specification for single tumor versus second tumor samples produced similar results with p < .001 and an odds ratio of 3.49. In other words, there appears to be a latent factor that is present in those who have a single tumor that is not present in those samples with a second primary tumor.

Furthermore, another SEM model was run combining the above two analyses so that the two distinct latent variables were used to predict disease status. The estimation of disease status was done via multinomial logistic regression. This was done because when the models were combined there were three levels of disease. In a multinomial logistic regression model, one level (in this case single tumor samples) was set as the reference group. Then n-1 logistic separate regression equations are run; where n is the number of levels of the dependent variable. Therefore, since each latent variable was regressed on disease status, there were two logistic regression equations run. Both latent variables predicted their respective disease status with p < .01. Again, this suggests that normal, single tumor cancer and second tumor cancer samples are separated by different sets of latent variables.

The first set of SEM models provided an initial proof of concept for full SEM models. This laid the groundwork for the more interesting question of what the underlying structure looks like for unique parts of the immunosignatures. Since further investigations are meant to look at the overall differences in immunosignatures as a whole, it is hypothesized that the latent factors differentiating groups are specific antibodies present in the sera samples; as explained above. Two experimental tests were conducted: a series of structural equation models and an examination of the peptide means across groups.

### SEM Models of Significant Peptides and Antibodies

**Figure 3:**
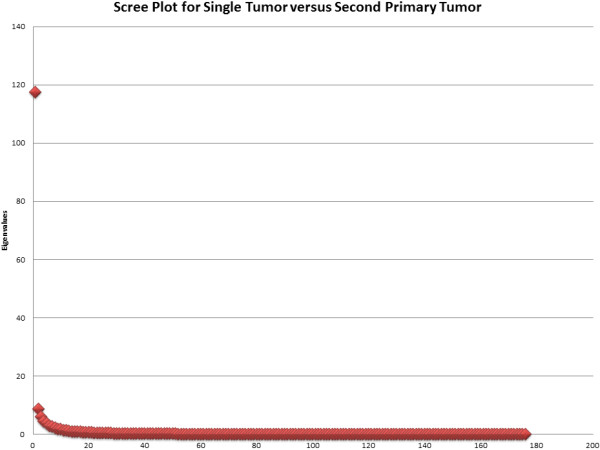
**Scree Plot for Single Tumor versus Second Primary Tumor**.

Next, all of the peptides that were statistically significant after a Bonferroni correction in the normal versus single tumor and second tumor versus single tumor contrasts were selected for further analysis. Following the same pattern as before, exploratory factor analysis models were run to determine how many underlying factors appeared to be present. This was done because selecting the top peptides might yield more than one factor; suggesting more than one antibody. For the normal versus single tumor contrast there were 176 peptides that were significant and there were 30 significant peptides for the second tumor versus single tumor contrast. The eigenvalues and scree plots suggest a three factor solution for the normal versus single tumor contrast and a one factor solution for the second tumor versus single tumor contrast. A scree plot for the second tumor versus single tumor contrast is shown in Figure 3. In other words, for the normal versus single tumor, the hypothesis is that there are three antibodies that differentiate the groups while there is only a single antibody differentiating the second tumor versus single tumor groups.

In the second tumor versus single tumor contrast, factor loadings from exploratory factor mixture models and Promax EFA models confirm an unstable second factor. This is because the loadings on the second factor are generally low and minimally larger than the first factor loading on the same peptide. Additionally, a two factor solution produced Heywood cases in which there were communality estimates greater than one; suggesting a problem with the two factor model. When single factor models were run, all of the peptides loaded highly on the one factor. As a result of the EFA models suggesting a single factor solution, a full SEM model was run in which all of the top 30 peptides were set to load on a single latent variable which was then regressed on disease status. In this model, the stable latent factor significantly correlated with disease status, p < 0.001. The odds ratio of 3.148 suggests that the single hypothesized antibody confers significant risk for acquiring a second tumor. Also, the means for all of the peptides in the second tumor samples were lower than the means for the single tumor samples. This suggests immune suppression. In other words, there appears to be an antibody present in samples with a single tumor that is not present in samples with a second tumor.

The normal versus single tumor samples is a bit more complex. A full SEM model containing all three hypothesized factors was unable to be estimated because there were more peptides than samples. Therefore, there were not enough degrees of freedom to run a full model containing all 3 groups. As a result, subsets and individual factors were tested individually. When tested individually, all of the three factors/hypothesized antibodies significantly correlate with disease, p < 0.01. Two of the latent factors positively correlated while the third negatively correlated with disease status.

Within the 176 significant peptides for normal versus single tumor samples, 162 peptides increase or have a higher mean in the cancer samples than in the normal samples increase. Conversely, 14 decrease or have a higher mean in the normal group than in the cancer group. In other words, there appears to be two new antibodies present in cancer samples not present in normal samples and one antibody present in normal samples that is not present in cancer samples. Immunosignatures are unique in analysis of the humoral response in that they can detect decreases in reactivity relative to normal levels.

One finding of particular note is a high covariance between the two positive factors (or proposed antibodies present in cancer that are not in normal samples). The high covariance and multicollinearity suggests that the two are very similar. When regressing both of the positive latent variables on disease, in every instance, only one of the latent factors was significant with p < .05. This is likely due to the way in which multiple regression partitions variance. In a multivariate regression model, the effect of one variable (x) is the unique contribution of that variable with all others held constant. Because there is so much common or shared variance, a vast majority of the variance is used up or accounted for by the first factor, not leaving enough unique unexplained variance left for the second factor to be significant as well.

A two level measurement model was run to test to see if the two factors were measuring a similar underlying construct. In this model, the two latent factors were set to load on a third latent variable. The theory behind this test was that if the two latent factors loaded on a single second level latent factor then the two original factors would be measuring the same underlying construct. One way this could occur is if the antibody had a highly complex structure. However, this model was not significant, RMSEA = .21, SRMR = .09. This suggests that the two factors are unique albeit highly similar.

There are a number of potential interpretations of this result. One of the more plausible biological hypotheses is the presence of subpopulations. Among two different cancer subtypes of single tumor breast cancer, there are likely two distinct antibodies; one for each subtype. If subpopulations are present in the data, it seems plausible that these two antibodies are quite highly similar because in the end they are still responding to breast cancer. The variations that lead to different subtypes may in fact be what makes the two positive latent factors separate and distinct from one another. The high covariance and multicollinearity may be a function of the fact that the two different subtypes are still breast cancer. The multicollinearity may be because they vary together, not that they have a similar sequence and see the same antigen. If two different antigens consistently arose in a tumor they would raise antibodies that varied together in samples but would see totally different antigens.

A second possibility is that this is modeling different times in the disease progression. As disease progresses it is likely different antigens are presented by the tumor to the immune system. If so, the relative amount of particular marker antibodies will also change.

## Discussion

We have explored a number of statistical models for analyzing immunosignatures. Each method explored herein helps answer a different research question relating to the analysis of immunosignatures. Descriptive statistics about an immunosignature can provide high level information about the general immune response in a signature. Exploratory factor analytic models (PCA and EFA) can be useful for classifying immunosignatures into different disease groups without any clinical information. CFA models can classify samples onto specified factors and could be developed into a useful model for determining the disease status of a single sample. As an extension, SEM models find some interesting and robust latent factor structure to immunosignatures which warrant further investigation.

### Implications of SEM Models

Latent factors can be reliably extracted from immunosignatures. These latent factors are clear, consistent and replicable patterns which differentiate disease state in a statistically significant fashion. At the very minimum, these latent factors can serve as strong biomarkers for disease. Given the design of the technology and the fact that antibodies are binding to peptides on immunosignature arrays, it is highly plausible that the latent factors are modeling individual antibodies.

Although future research is needed to conclusively confirm the relationship between modeled latent factors and antibodies, the potential of having a high-throughput bioinformatics-driven method for antibody discovery creates countless potential avenues for future applications. The primary benefit of this methodological approach is to reduce the time it takes to identify antibodies associated with various clinical situations. Doing so will reduce cost and increase the speed of advancement in biomedicine. Additionally, the reduced cost and speed may open doors that were beyond the realm of consideration just a short time ago. For example, a method for quickly and inexpensively detecting an antibody could play a crucial first step in developing personalized vaccines.

Below we present a multi-step procedure for detecting latent factors and potentially antibodies in an immunosignaturing study. The first step is to run an exploratory factor analysis on the data with rotation. Various rotations can be explored but Promax or Geomin are recommended. EFA models are a useful starting place for multiple reasons. First, it ensures that the groups are different constructs and significantly different from one another. This determination can be made by looking at scree plots and eigenvalues to assess the probable number of groups in the model; which should be equal to the number of known disease states. The samples should load correctly on a given factor with a high classification rate.

At this point, cross loadings in an EFA model can be investigated. If clinical data exists, it would be of use to try to assess if there are potential reasons for why a specific sample may be cross loading. For example, is there a history of cancer in a normal sample that cross loads on a cancer sample which might suggest the person is in a transition phase? This may be a way of detecting aberrant cases or outliers. That said, haphazardly removing cases from a dataset is NOT advocated in any fashion. Cross loadings were not analyzed in this paper due to a lack of additional information and clinical data upon which to draw any relevant conclusions.

From here, an appropriate test statistic comparing the groups can be run on the peptides in order to test for statistical significance. T-Tests or logistic regression and their multivariate extensions ANOVA and multinomial logistic regression are a few potential methodological tools. The specific test should be picked with respect to the features of the data being analyzed. For example, in this paper, we used a Satterthwaite corrected T-Test because of unequal samples sizes and variances. A correction should be made to protect against alpha inflation. Although a number of tests exist for this purpose, the Bonferroni correction is among the most common; even if it may be somewhat conservative.

A traditional EFA model or an exploratory factor mixture model can be used to infer the structure of the significant peptides within each group. This information can be used to create a full structural equation model. However, as part of good model building practices, starting with a CFA measurement model is recommended; especially because the iterative nature and complexity of these models may lead to convergence problems. Additionally, information from these simpler models can be used to specify starting values in full SEM models if convergence problems occur. CFA measurement models specify which peptides load together on a given latent factor. Checking the fit of the measurement models can confirm the accuracy of the model. However, given that CFA is so similar to EFA methods, it is unlikely that differing results would be obtained.

Once a working measurement model has been obtained, a full SEM model can be created by regressing the latent factors on disease state. It is important to test a full SEM model for a number of reasons. Although EFA and CFA models may suggest that a group of significant peptides are related in some way, without a full SEM model, there is no way of knowing whether the relationship is a significant predictor of a specific disease state. In the absence of predictive validity for a specified disease state, any relationship among the peptides is trivial and would not suggest that it is because of a common antibody. The same conclusion can made if the latent factor is predictive of disease states beyond the hypothesized state.

If a significant SEM model can be obtained, wet lab validation can then attempt to determine if the model is correct. One potential way of testing this in the wet lab would be to use the designated peptides to affinity purify the antibody from the sera. The prediction is that the different peptides would purify the same antibody. This could be tested by immunosignaturing the antibodies purified.

### Screening and Presymptomatic Screening for Disease

The relative ease from which samples can be classified and differentiated with all of the methods explored herein makes this technology an excellent use for disease screening. Whether examining the loadings of new samples in a CFA model or as part of a larger SEM model, this technology can allow researchers to screen patients in a variety of contexts. This initial research suggests that immunosignaturing could be developed into a quick and inexpensive method of screening for cancer. Taking a small sera sample from an individual is much less expensive and intrusive than traditional screening methods such as mammograms. One early potential use for immunosignaturing would be to help follow at risk populations; such as those individuals with a family history of cancer. Immunosignatures could be taken at regular intervals between regularly scheduled mammograms. If the generated immunosignature from an interim test started to suggest a closer similarity to cancer, this could prompt physicians to follow the patient more closely or advise additional screening. Immunosignatures could be used in the same way for individuals who already have cancer. In this case, if an immunosignature suggested the person was developing an antibody signature indicative of a second tumor (or more closely loading on a latent factor biomarker), the individual could be followed more closely to detect the presence of a second primary tumor.

Screening for a specific disease state is fairly straightforward. A well curated collection of disease samples would form baseline control factors. A sera sample would be taken from an individual and their sample would be allowed to freely load in a CFA model across relevant disease conditions. A significant loading on a disease factor would provide strong evidence for the person having a given disease.

There are a number of ways in which a presymptomatic screening test could be developed from immunosignatures. This could be done by collecting a longitudinal or time series sample of sera from an individual and following the factor loadings on a disease state over time. As the loadings on a disease factor tend to increase the individual could be watched more closely and additional screening for a disease could be recommended by a physician. A number of statistical methods and time series analyses such as latent transition analysis (LTA) could be employed to model this.

## Conclusion

Immunosignaturing is a novel approach for understanding disease. A number of statistical methods including, exploratory factor analysis, confirmatory factor analysis, descriptive statistics, corrected t-tests, ANOVA, logistic and multinomial logistic regression, mixture models and structural equation modeling have shown promising abilities for analyzing different dimensions of immunosignatures. Immunosignaturing in the context of breast cancer has been shown to be a good platform for differentiating groups of samples based on disease status, determining the disease status of specific samples as well as potentially serving a role in the discovery of antibodies for specific diseases.

Despite many new challenges posed by immunosignaturing microarrays such as competitive binding and binding to multiple sites, the analyses conducted here clearly illustrate the usefulness of classical analytical methods to produce accurate results. The results are particularly noteworthy because of the lack of structure in the data and lack of a full pattern matched experimental design. The early results of structural equation modeling are very promising. Although wet lab validation is needed for the proposed methodology of antibody discovery, even if the latent factors turn out not to be a specific antibody, the model can still serve as an excellent biosignatures for disease screening.

Early detection of cancer is among the best predictors of survival. Continued development of immunosignaturing into a screening and presymptomatic screening diagnostic tool will aid in early discovery and help turn the corner in the fight against cancer. Future research in this field should aim at validating the hypothesis that the latent factors modeled here are in fact antibodies and to develop the technology into a diagnostic screening tool.

## Competing interests

The authors declare that they have no competing interests.

## Authors' contributions

JB helped with design of methods, statistical analyses and drafting of the document. PS helped with collection of biological samples, design of methods, statistical analyses and drafting of document. SJ helped with collection of biological samples, design of methods and drafting of document. VD helped with design of methods, statistical analyses and drafting of document. All authors read and reviewed the final manuscript.
